# The impact of economic recession on maternal and infant mortality: lessons from history

**DOI:** 10.1186/1471-2458-10-727

**Published:** 2010-11-24

**Authors:** Tim Ensor, Stephanie Cooper, Lisa Davidson, Ann Fitzmaurice, Wendy J Graham

**Affiliations:** 1 Immpact, School of Medicine and Dentistry, University of Aberdeen, Aberdeen, UK

## Abstract

**Background:**

The effect of the recent world recession on population health has featured heavily in recent international meetings. Maternal health is a particular concern given that many countries were already falling short of their MDG targets for 2015.

**Methods:**

We utilise 20^th ^century time series data from 14 high and middle income countries to investigate associations between previous economic recession and boom periods on maternal and infant outcomes (1936 to 2005). A first difference logarithmic model is used to investigate the association between short run fluctuations in GDP per capita (individual incomes) and changes in health outcomes. Separate models are estimated for four separate time periods.

**Results:**

The results suggest a modest but significant association between maternal and infant mortality and economic growth for early periods (1936 to 1965) but not more recent periods. Individual country data display markedly different patterns of response to economic changes. Japan and Canada were vulnerable to economic shocks in the post war period. In contrast, mortality rates in countries such as the UK and Italy and particularly the US appear little affected by economic fluctuations.

**Conclusions:**

The data presented suggest that recessions do have a negative association with maternal and infant outcomes particularly in earlier stages of a country's development although the effects vary widely across different systems. Almost all of the 20 least wealthy countries have suffered a reduction of 10% or more in GDP per capita in at least one of the last five decades. The challenge for today's policy makers is the design and implementation of mechanisms that protect vulnerable populations from the effects of fluctuating national income.

## Background

What effect is the current worldwide recession likely to have on the health of populations, particularly those in the least wealthy countries? This key question has featured strongly in international meetings such as the G8, and as a focus of several initiatives, including the High Level Taskforce for Innovative Financing [1]. Prior to the present economic slump there was already considerable concern that many countries were failing to have a substantial impact on basic health outcomes [2]. In particular, many of the least wealthy countries are essentially off-track to achieve the 2015 targets for child and maternal health (Millennium Development Goals 4 &5) set by the international community in 2000 and to be reviewed intensively this year. A core policy question is what governments in aid-receiving and donor-countries can do to mitigate the effect of the current recession. Will any mortality gains be reversed, for example, or will the opportunity cease to protect vulnerable groups and so accelerate progress? This paper explores such questions through examination of an historic dataset assembled on maternal and infant mortality for selected rich and middle income countries spanning most of the twentieth century and a period of both recession and growth.

### Health outcomes and incomes

The study used GDP per capita as a general descriptor for the state of the economy. Recession is generally defined as two quarters of negative economic growth. The impacts are clearly felt beyond a change in income in particular on levels of employment, poverty and unequal effects on different socio-economic groups. GDP per capita does not fully account for all these influences but provides a useful summary variable for the general health of the economy that is available universally and consistently available for most countries unlike, for example, unemployment and poverty rates where local definitions and conventions vary widely.

**Figure 1:**
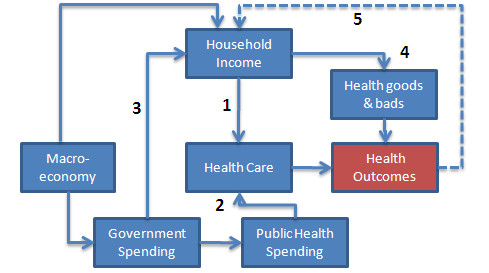
**Health and wealth: simplified causal relationships**. Various routes through which recession may impact on health outcomes. The numbers in this figure link to textual references.

The link between individual income and health outcomes is well established at an individual level. At a national level, whilst it is self evidently true that poorer countries generally have inferior health status, the link between changes in income and health outcomes is less clear cut. There are a number of routes through which recession may impact on health outcomes (Figure 1). Household income is directly affected, which in turn affects a household's ability to cover out of pocket costs of services (1). In systems that are free at point of delivery these are mainly 'demand-side' costs: transport, time taken away from work. The potential effect is larger in systems where a user must also contribute to the cost of service through a user charge.

In addition, spending on health care services is determined by the State's ability to raise funds through taxation, social insurance or other means. This in turn affects the ability of a national government to properly finance basic services (2). A recent study of progress towards the Millennium Development Goals (MDGS) found that the direct influence of GDP on health was positive but relatively modest [3]. At the same time the effect of increased human resources made possible by greater spending was strongly associated with progress towards the MDGs. The combined influence of changes in income through the effect on households and public spending is therefore substantial.

Recession also impacts on the State's ability to fully finance transfer payments such as pension and unemployment benefits, which in turn affect health improving expenditures (3). A sharp decline in the real value of state pensions in Russia during the late 1990 s, for example, was associated with a reduction in the use of health services, protein consumption and life expectancy amongst pensioners [4].

The negative effect on spending may be offset somewhat if a government chooses to protect basic health services at the expense of other commitments. It is thought, for example, that in Cuba preferential access given to services for women and children may have helped to protect basic health outcomes from the worst effects of the US embargo [5]. Assistance from donors, which finances a substantial part of the health sector in many developing countries, could also ease the impact, although worldwide recession might distort these flows.

A Russian study suggests that health is also affected positively or negatively by consumption of other goods and behaviours (4). In the United States, data suggest that recessions tend to lead to reductions in fertility [6]. This study indicates that this effect is asymmetrical as increasing unemployment makes poorer families more cautious about having children. Since these households consistently have higher maternal and child mortality than the rest of the population, there was an associated improvement in average health outcomes. Similarly, a study in post-war Japan found that deaths commonly associated with poor lifestyles and the consumption of 'health bads' such as poor diet or tobacco use are pro-cyclical - in other words, they tend to increase when the economy improves [7]. Similar evidence was reported for the United States with smoking and obesity related disease in particular increasing when the economy strengthens [8].

The relationship between health and income is thus not uni-directional nor consistent across time or settings. Better health can drive improvements in livelihoods and contribute to increased national wealth [6]. There is evidence, for example, that improvements in life expectancy have a positive effect on GDP growth rates, and that improved maternal and infant health improves the economic position of households [9,10]. Conversely, differences in growth rates between Africa and Asia have at least partly been attributed to differences in underlying health outcomes [11]. There is well established evidence that the relationship between income and health outcomes is more important for countries at an earlier stage of development [12]. This suggests that the impact of economic shocks will also change (weaken) as the economy grows.

One of the problems with assessing the effect of recession is assembling a sufficient time series of data on health outcomes. A recent study took advantage of the substantial dataset provided by the Demographic and Health Surveys, a standard survey methodology examining reproductive and child health and now applied in more than 75 countries, to examine the association between economic growth and infant mortality across 59 countries. The study found a substantial negative effect of per capita income on infant mortality [13]. In this paper we use a time series assembled from records dating back to the early 20^th ^century in order to examine the effect of income on infant and maternal mortality.

## Methods

**Figure 2:**
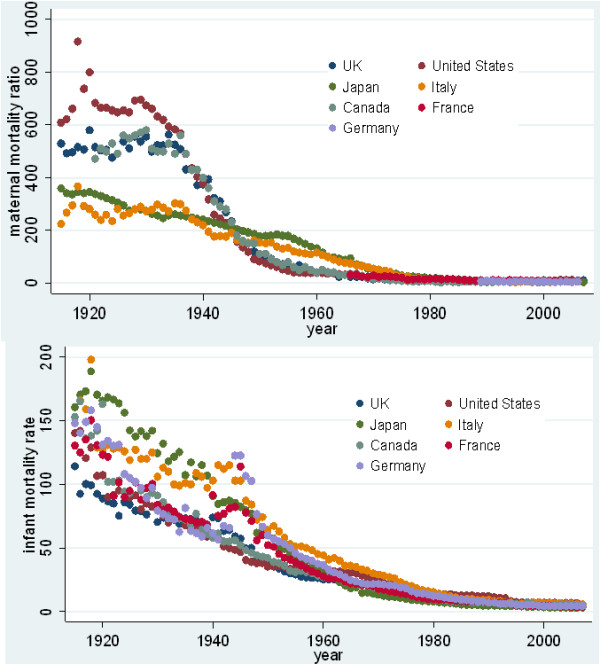
**Long run trends in maternal mortality**. Graphical representation of substantial negative association between long run GDP per capita and mortality.

The analysis focuses on 14 high and middle income countries: high income countries were United Kingdom, United States, Japan, Italy, Canada, France, and Germany; low/middle income countries were Argentina, Brazil, Malaysia, Mexico, Nicaragua, Russia and Thailand. The time series for each country was assembled from a variety of historical sources. For high income countries data were obtained from as early as 1915, but records from this time until the mid-1930 s are patchy and inconsistent, resulting in implausible estimates (as seen in Figure 2 below). The series for these high income countries as a whole appears to become more consistent from 1936 and this year is, therefore, used as the starting point for the analysis reported in this paper. Regressions were undertaken using data for the earlier period but, not surprisingly given the quality of the data, there were no statistically significant associations. For most of the low/middle income countries, data are not available until the 1950 s or later. Per capita income (Gross Domestic Product) was obtained for the same period using the Maddison database [14]. The data set permits an examination of the long run effects of GDP fluctuations on maternal and infant mortality. Data were extracted from various sources e.g Demographic Year Books, and were also received directly from contact with the National Statistics Offices (see acknowledgements). Estimates of the maternal mortality ratio - the most commonly used measure (maternal deaths per 100,000 live births) used here only includes direct obstetric causes of death since this enables the longest consistent time-series to be viewed across the selected countries. Indirect causes were not included into most national routine statistics of maternal deaths until after the mid-1960 s.

The substantial negative association between long run GDP per capita and mortality is borne out both by graphical representation (Figure 2) and simple regressions of outcomes (maternal mortality ratio (MMR) and infant mortality rate (IMR)) on GDP per capita (log values) (Table 1). The association with income appears to vary over the years, with the strongest correlation in the 1966 to 1980 period for MMR and 1981 and later for IMR.

**Table 1:** Association between health outcomes and GDP per capita

	1936 to 1950		1951 to 1965		1966 to 1980		1981 and later		All years		All years	
	**High income economies**										**Low/Middle income**	

	**Coefficient**	**t-stat**	**Coefficient**	**t-stat**	**Coefficient**	**t-stat**	**Coefficient**	**t-stat**	**Coefficient**	**t-stat**	**Coefficient**	**t-stat**

MATERNAL MORTALITY RATIO												

ln_gdp	- 0.739	-3.09	- 1.205	-8.77	- 3.036	-17.01	- 1.095	-5.86	- 2.287	-48.5	- 1.910	- 42.70

Constant	11.707	5.78	14.913	12.27	31.588	18.8	12.602	6.85	24.552	57.11	20.130	- 54.80

INFANT MORTALITY RATE												

ln_gdp	- 0.284	-3.16	- 1.037	-32.24	- 1.604	-27.02	- 1.895	-30.45	- 1.575	-105.26	- 1.099	-29.05

Constant	6.613	8.75	12.686	44.67	17.929	32.1	20.509	33.51	17.495	129.54	12.803	41.92

The rich country group appears to divide into two distinct sub-groups with regard to maternal mortality. In both Italy and Japan lower maternal mortality is reported from the beginning of the time series, yet their infant mortality remains comparable to other countries. Levels of maternal mortality in the UK, United States and Canada are reported as substantially higher in the 1920 s and early 1930 s and it is only from the late 1930 s and mid-1940 s that levels fall substantially to those at or below those reported in Japan and Italy. The dramatic decline found here in the UK, USA and Canada is consistent with other published series, such as the work of Loudon [15].

Long run time trends mean that any association between the variables using untransformed data will tend to be dominated by the trend itself rather than explaining the effect of deviations from the trend produced by recession or temporary booms. Our paper thus follows other studies of the effect of the macro-economy by transforming outcomes and GDP in order to focus on short term fluctuations [7,13]. A first difference logarithmic transformation regresses the change in mortality outcomes on changes in GDP per capita (constant prices). Fertility is strongly associated with maternal and infant deaths and so is an important potential confounder. The crude birth rate is added to proxy this effect (the total fertility rate is not available for all observations in the data set). Lagged (logged) values of the mortality and income variable are also included. The format is specified as follows:

$$lnH_{ct}-lnH_{ct-u}=\beta_{0}+\beta_{1}\left(lnGDPPC_{ct}-lnGDPPC_{ct-u}\right)+\beta_{2}lnGDPPC_{ct-u}+\beta_{2}H_{ct-u}+\beta_{4}CBR_{ct-u}$$

Where *H_ct _*is the health status variable of interest at time t and country c, *GDPPC_ct _*(time t, country c) is GDP per capita in real (2000) US dollars, CBR is the crude birth rate and *u *is the lag. A lag of five years is used for all models. The reason is that mortality data are mostly available at five year intervals. Whilst it is possible to impute values between these years, there is no possibility of picking up genuine fluctuations in mortality on a more regular basis. The use of a fixed lag is less restrictive than it seems: in the context of this model, a lag means that the cumulative five year change in GDP is expected to have a cumulative five year association with health outcomes.

We investigate the dataset in two stages. First, both fixed and random effects panel regression were used to explore the effect of changing per capita income on maternal, infant and neonatal mortality. A Hausman test, used to test the consistency of random versus fixed effects, was statistically significant (P < 0.01), implying that random effects are inconsistent and a fixed model should be used for both the upper and middle/low income country groups. Separate estimation was carried out for the (now) upper income countries and low/middle income countries. The dataset for the upper income countries was divided into fifteen year time periods: 1936 to 1950, 1951 to 1965, 1966 to 1980 and 1981 to 2005. The data for low/middle income countries is not divided by periods because of the small number of observations available for the earlier time periods. One of the potential problems with this specification is simultaneity bias if gdp per capita both determines maternal and infant outcomes and is affected by them. The use of change variables should minimise this risk. An instrumental variable (xtivreg in Stata) method that mitigates simultaneity leading to inconsistent estimates was also estimated. A time trend and total fertility rate were used to instrument the change in GDP variable. A Hausman test was also used to compare specifications and test for inconsistency. The tested was rejected (p > 0.5) for both low/middle and upper income groups suggesting no evidence of simultaneity.

A second stage of modelling was to focus on each country individually and the differences in association between per capita income and health during the four post-war time periods. Statistical tests - Durbin Watson (DW) and Breusch-Pagan - indicated that ordinary least squares regression is inefficient as a result of heteroscedastic and autocorrelated (lag 1) errors. Durbin Watson statistics all indicated positive serial correlation with the statistic below the lower bound of significance (Statistics as follows: United Kingdom 0.86, United States 0.26, Japan 0.86, Italy 1.14, Canada 0.98). A generalised least squares model with robust standard errors was therefore used (Prais-Winsten and Cochrane-Orcutt error correction regression, specified as 'Prais' in Stata v10.1). Post-estimated DW statistics suggested that the correction had successfully eliminated serial correlation from the estimation for Canada, Italy and Japan and reduced for United States and UK (the statistic was in the zone of indeterminacy).

## Results

A negative association (significant at 5% level) between changes in income and outcomes was found for both maternal and infant mortality (Table 2). Estimation across the four periods suggests that the absolute effect of short run fluctuations in per capita income declines over time. A significant negative association between changes in income and both maternal and infant mortality is indicated in the first two periods (1936 to 1950, 1951 to 1965). The absolute impact is similar since whilst the coefficient is larger in the second period the mean value is lower. In both periods a 10% reduction in income implies an increase in the MMR of just over six deaths per 100,000 live births. In subsequent periods (1966 to 1980, 1981 to 2005) no statistically significant association is detected. Likewise, for infant mortality in the first two periods a 10% reduction in income is associated with a statistically significant (1% level) increase in IMR of around 3/1000 live births. For neonatal deaths a significant negative effect is only found in period two (1951 to 1965), although the paucity of data for the first period reduces the statistical power of the regression.

**Table 2:** Associations between health outcomes and changes in GDP per capita

	Upper income countries										Middle/low income countries All years	
	**1936 to 1950**		**1951 to 1965**		**1966 to 1980**		**1981 to 2005**		**All years**			

	VARIABLE MEANS											

MMR	257		83		25		7		70		181	

IMR	71		35		18		7		29		56	

NNMR	30		19		12		4		13			

GDP per cap	5,089		7,347		12,412		19,009		12,210		3,957	

	MATERNAL MORTALITY RATIO											

	Coefficient	t-stat	Coefficient	t-stat	Coefficient	t-stat	Coefficient	t-stat	Coefficient	t-stat	Coefficient	t-stat

Change GDP	- 0.22	- 2.95	- 0.73	- 2.30	- 0.93	- 1.56	- 0.06	- 0.10	- 0.22	- 2.11	- 0.77	-4.93

GDP-5	- 0.70	- 5.21	- 0.46	- 3.24	- 1.85	- 5.75	0.14	0.48	- 0.62	- 9.05	- 0.26	-1.99

MMR-5	0.21	2.53	- 0.39	- 8.65	- 0.80	- 6.92	- 0.70	- 8.24	- 0.25	- 11.39	- 0.22	-3.48

CBR-5	0.37	1.58	0.19	1.02	1.14	4.69	1.12	2.85	- 0.25	- 2.41	0.14	2.63

Constant	3.15	2.35	4.94	2.60	16.32	4.80	- 3.00	- 0.83	7.01	7.95	2.49	1.79

Observations	75		75		85		164		399		212	

Groups	5		5		6		7		7		7	

F statistic		29.02		27.38		16.06		24.93		35.45		10.91

Prob > F		<0.001		<0.001		<0.001		<0.01		<0.01		p < 0.01

	INFANT MORTALITY RATE											

Change GDP	- 0.36	- 4.21	- 0.64	- 5.62	- 0.01	- 0.03	- 0.05	- 0.31	- 0.41	- 11.04	- 0.20	-2.43

GDP-5	- 0.57	- 3.93	- 0.53	- 6.97	- 0.35	- 1.96	- 0.99	- 6.09	- 0.26	- 6.56	- 0.15	-3.65

IMR-5	- 0.56	- 3.79	- 0.57	- 9.94	- 0.38	- 3.00	- 0.51	- 8.39	- 0.18	- 6.62	- 0.11	-3.68

CBR-5	0.85	3.60	0.05	0.71	0.43	7.87	- 0.46	- 5.32	0.06	1.67	- 0.04	-0.85

Constant	4.53	2.91	6.48	6.68	2.96	1.41	11.72	6.38	2.63	5.72	1.63	3.37

Observations	104		105		105		182		496		294	

Groups	7		7		7		7		7		7	

F statistic		11.39		39.35		20.92		44.76		33.39		26.56

Prob > F		p < 0.01		p < 0.01		p < 0.01		p < 0.01		p < 0.01		p < 0.01

Lack of data precludes a separation of results by period for the low/middle income country group. Pooled estimations for maternal and infant outcomes suggest a significant negative effect of income (Table 2). A 10% reduction in real GDP per capita is associated with an increase in the MMR of 16 (8.5%) and IMR of 1.4 (2%); equivalent to an additional 13,500 infant and 1,500 maternal deaths across the seven low/middle income countries included in the study.

**Figure 3:**
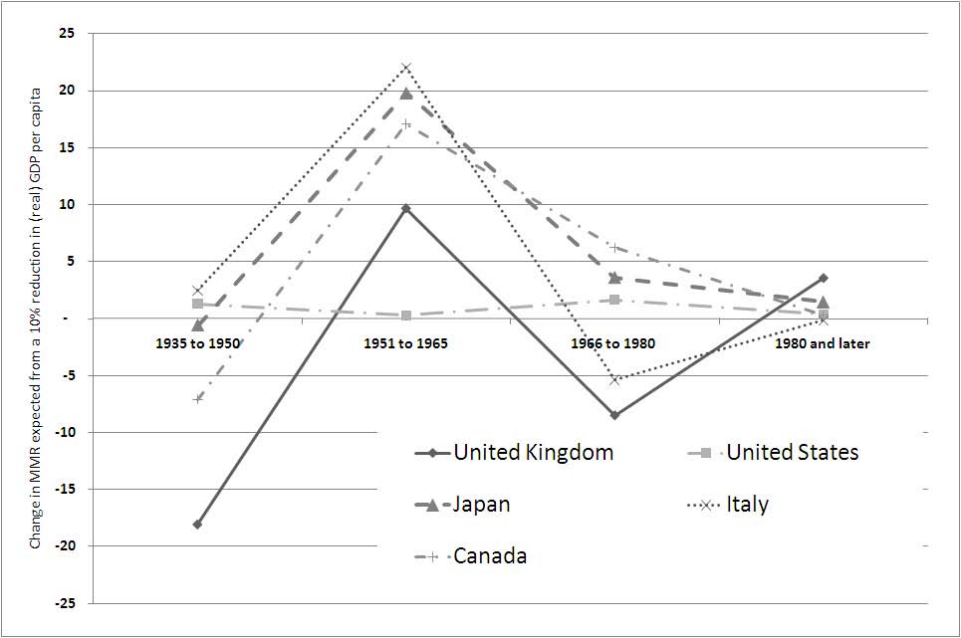
**Changes in MMR associated with 10% reduction in (real) GDP per capita**.

**Figure 4:**
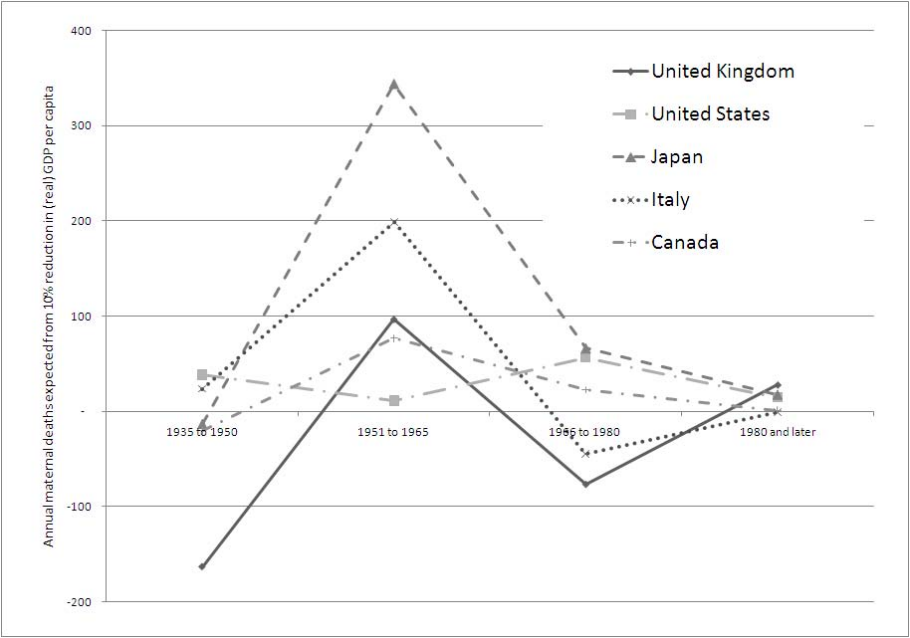
**Annual numbers of maternal deaths associated with 10% reduction in (real) GDP per capita**.

Whilst the associations explored in this pooled analysis are statistically significant, the magnitude of the effects is relatively modest. The data series (Figure 1) suggest that patterns of decline in mortality, and maternal mortality in particular, differ between countries. This is indeed confirmed in the individual country regressions. The data imply a substantial but varying association with MMR (Figure 3 &4). A number of patterns can be discerned. Firstly, for two countries, Japan and Canada, substantial and statistically significant increases in maternal mortality coincide with GDP decline in the 1950 to 1966 period. This relationship was found to continue from 1966 to 1980, although it only remains significant for Japan. Interestingly these two countries exhibited similar trends and levels of maternal mortality by the 1950 s, but for the earlier periods had rather different trajectories and indeed continued to experience different fertility profiles almost to the end of the observed period - 1980. The second type of pattern is one of inconsistency and is demonstrated by the United Kingdom and Italy. In two periods the association is pro-cyclical, thus a decline in income leads to a reduction in maternal deaths (significant only for the 1966 to 1980 period). Taking all periods together, the relationship is not statistically significant. The third and final pattern is one characterised by a consistent lack of effect on maternal mortality of fluctuating income, as seen in the United States series. There are several possible explanations for this apparent insulation against economic shocks. One is that the country's economy has reached the stage whereby prioritisation of maternal health care is not, at the margin, affected by changes in income. A further possible explanation is the adjustments mentioned by Dehejia et al [16], whereby families reduce births during periods of recession. This impacts disproportionately on poorer women, who also tend to have inferior maternal health outcomes and, as a consequence, the overall level of maternal mortality falls. Interestingly, the picture for infant mortality is somewhat different from all three patterns regarding maternal mortality. In the US, for example, a substantial and statistically significant effect on IMR was associated with falls in income.

## Discussion

There is evidence to suggest that income is becoming less important as a determinant of mortality outcomes today than it was in the past [17]. Technological gains that historically took decades to achieve and to scale-up in now rich countries are potentially available at much lower cost to countries and at an earlier stage of development. This effect is borne out by the work of Kenny and others that suggest that while infant mortality has consistently fallen worldwide since 1900, the link to income growth is weakening [17]. Better average health outcomes would thus appear to be achievable in the modern era at lower average income levels. Yet the experience of the 14 countries in the sample in this paper suggests overall trend improvements in income only tell part of the story. Fluctuations from the trend as the economy goes through recession or growth is also associated with basic health outcomes like mortality. This was true for the high income countries in our series just before and soon after the Second World War and also appears to hold for the low/middle income countries in the 1960 s and 1970 s.

Furthermore, there seem to be differences in the experiences of the countries in the group. Since the Second World War, economic cycles appear to have a substantially greater association with maternal mortality in Japan, Italy and Canada than in the United States or the United Kingdom. The reasons for these differences are not entirely clear. In the United Kingdom, for example, it could be conjectured that the introduction of the National Health Service ensured financial access in good times and bad, and for all types of health problems. Yet similar risk pooling was not and still is not available in the United States for all conditions, despite the introduction of Medicaid and Medicare in the 1960 s. The US system does, however, provide free Emergency Room (Emergency Medical Treatment and Active Labor Act, 1986) care which will include emergency complications during acute pregnancy and childbirth. There is also some evidence that families in the United States and in other developed economies make adjustments to their expectations about family size in response to economic cycles. This 'feedback' may, in turn, help to minimise adverse effect on health outcomes, particularly amongst the poorest groups. There is good historical and contemporary evidence that the burden of maternal mortality within developed and developing countries is borne disproportionately by the poor [17-19]. The national data used in our analysis necessarily masks differential effects of recession on marginalised groups but it is reasonable to expect that much of this effect would be on the poorest groups for which there would be much larger changes in mortality. This key issue has major implications for equity goals, and would benefit from further investigation at a country level, including changes in uptake of care in the face of recession.

Substantial reductions in GDP per capita in richer countries are rare. Recession is generally defined as negative growth for two or more consecutive quarters, and negative growth of more than 5% is unusual [20]. Yet in the developing world negative growth remains common. High population growth means than even zero or modest positive overall growth still leads to declining per capita incomes. According to World Bank data, 18 of the 20 poorest countries suffered a 10% reduction in GDP per capita, eight suffered a 20% reduction, and six a 30% (or more) reduction in at least one of the last five decades (based on 2007 GDP per capita and excludes Zimbabwe, North Korea and Palestine due to lack of data). The models presented in this paper suggest that such reductions could have a substantial effect on maternal and infant mortality in these countries. The likelihood of such effects becoming a reality is supported by other studies particularly that of Baird and colleagues [13] for infant outcomes, and highlighted in recent international documents [1]. Studies in Thailand and Indonesia suggested that the 1997 economic crash had a significant impact on spending by households on health care [21,22]. These studies reinforce the need for public health action to help ensure a safety net for the poorest who are most vulnerable to such shocks.

The limitations of the analysis presented here inevitably arise from the data, both in scope and quality. Interpretation of long time series of mortality and birth data -over 70 years for some countries - must acknowledge the risk of artefact effects owing to changing definitions and completeness and quality of data capture, problems which are further compounded by cross-country and pooled analyses. We have endeavoured to limit some of these risks by excluding data for the early years of the 20^th ^century which exhibit inconsistencies and implausible fluctuations. Finally, the processes and factors that affect maternal and infant health are likely to be different from those affecting other measures of health status. The results may not, therefore, be general to the health of other population sub-groups.

## Conclusions

The historical evidence presented in this paper yields conclusions that are necessarily speculative, but nevertheless suggest that health gains, particularly in countries at an early stage of development, are vulnerable to fluctuating GDP. Substantial additional maternal and infant deaths can be expected if the associations noted here for richer countries during the 20^th ^century are repeated for countries at similar stages of development today, particularly as many of these countries have considerably larger populations and much higher levels of mortality. The challenge for today's policy makers is the design and implementation of mechanisms that protect vulnerable populations from the effects of fluctuating national income [23]. Financing such systems is out of the reach of many developing countries using domestic resources alone and reinforces the need to maintain and even increase external development assistance during periods of economic crisis. Such assistance should be focused on improving systems rather than on traditional disease specific programme support. It is notable that health systems in richer countries, including most of the countries described in this paper, developed both the readiness to provide service when required and in the risk pooling function that ensures that financial barriers do not inhibit use of services. Most health programmes depend on a common system structure and recognising these inter-relationships is increasingly seen as an important part of a development assistance strategy [3]. These inter-relationships are well illustrated in the provision of maternal health services where well qualified staff must be supported by a strong infrastructure capable, for example, of ensuring referral, surgical delivery and a quality blood supply. This suggests that external assistance to support countries through recession and help them achieve goals such as the MDGs should be directed towards two main areas. Firstly, to strengthen systems for helping individuals and households cope with both supply and demand side costs of maternal care [24]. Secondly to assist countries to strengthen supply-side networks for supporting improved maternal health services and ensure that services are available when women present for service.

The current global challenge brought about by financial collapse comes at a time when progress towards achieving the MDGs requires speeding-up and scaling-up. The key question is whether the challenge can catalyse the invariably difficult decision of rationing scarce resources - here to protect mothers and infants.

## Competing interests

The authors declare that they have no competing interests.

## Authors' contributions

Paper conception: WJG, TE. Acquisition of data: SC, LD, AEF. Analysis and interpretation of data: WJG, TE, AEF. Drafting of manuscript: TE, WJG. Critical revision: TE, WJG, SC, LD, AEF. All authors read and approved the final manuscript.

## Pre-publication history

The pre-publication history for this paper can be accessed here:

http://www.biomedcentral.com/1471-2458/10/727/prepub
